# Enhanced CenterTrack for Robust Underwater Multi-Fish Tracking

**DOI:** 10.3390/ani16020156

**Published:** 2026-01-06

**Authors:** Jinfeng Wang, Mingrun Lin, Zhipeng Cheng, Renyou Yang, Qiong Huang

**Affiliations:** 1 College of Mathematics and Informatics, South China Agricultural University, Guangzhou 510642, China; wangjinfeng@scau.edu.cn (J.W.); chengzhipeng@stu.scau.edu.cn (Z.C.); 2 Southern Marine Science and Engineering Guangdong Laboratory, Zhanjiang 524000, China; youngrenyou@zjblab.com

**Keywords:** multi-object fish tracking, underwater aquaculture, multi-branch feature extraction, occlusion handling, MF25 dataset

## Abstract

Accurate monitoring of fish movement is essential for understanding behavioral patterns and group dynamics in aquaculture systems. Underwater scenes—characterized by dense populations, frequent occlusions, non-rigid body motion, and visually similar appearances—present substantial challenges for conventional multi-object tracking methods. We propose an improved CenterTrack-based framework tailored for multi-fish tracking in such environments. The framework integrates three complementary components: a multi-branch feature extractor that enhances discrimination among visually similar individuals, occlusion-aware output heads that estimate visibility states, and a three-stage cascade association module that improves trajectory continuity under abrupt motion and occlusions. To support systematic evaluation, we introduce a self-built dataset named Multi-Fish 25 (MF25), continuous video sequences of 75 individually annotated fish recorded in aquaculture tanks. The experimental results on MF25 show that the proposed method achieves an IDF1 of 82.5%, MOTA of 85.8%, and IDP of 84.7%. Although this study focuses on tracking performance rather than biological analysis, the produced high-quality trajectories form a solid basis for subsequent behavioral studies. The framework’s modular design and computational efficiency make it suitable for practical, online tracking in aquaculture scenarios.

## Simple Summary

Tracking multiple fish in underwater environments is essential for studying fish behavior and supporting ecological monitoring. However, poor visibility, complex backgrounds, and frequent occlusions make reliable tracking difficult in real underwater scenes. In this study, we propose an improved method for underwater multi-fish tracking based on CenterTrack. The proposed approach improves tracking stability and accuracy under challenging underwater conditions. Experimental results on underwater fish datasets show that our method performs more reliably than existing approaches. This study provides a practical tool for automated analysis of fish behavior in natural underwater environments.

## 1. Introduction

With the continuously growing global demand for aquaculture products, intelligent aquaculture has emerged as a key approach to achieving sustainable and efficient fishery production. In intensive aquaculture systems, large numbers of fish are cultivated in enclosed or semi-enclosed environments, making automated and non-invasive monitoring of fish movement, behavior, and group dynamics increasingly important [1,2,3]. Behavioral indicators such as swimming patterns, social interactions, and responses to environmental stimuli provide valuable information for welfare assessment, feeding optimization, and behavioral research [4,5,6].

Video-based monitoring using computer vision has shown considerable potential for extracting fine-grained movement trajectories and enabling quantitative analysis of fish behavior [7,8,9]. Accurate and continuous multi-fish tracking therefore serves as a fundamental technical component for a wide range of downstream aquaculture studies, including behavioral analysis and population-level monitoring. Although this work does not directly model biological or physiological indicators, reliable trajectory extraction is a necessary prerequisite for such higher-level analyses in both aquaculture engineering and fish behavior research.

Despite recent progress, underwater multi-fish tracking remains a challenging task due to complex imaging conditions, including uneven illumination, light refraction, water turbidity, and suspended particles [10]. In addition, fish exhibit non-rigid body deformation, rapid and abrupt motion, and highly similar visual appearances. High-density farming environments further exacerbate these challenges by introducing frequent occlusions and frequent target interactions, making robust identity association and long-term trajectory continuity particularly difficult [11,12,13].

Existing studies have explored a variety of vision-based approaches for fish monitoring and tracking. These include methods for estimating ventilation rates [14], swimming posture, and tail-beat frequency [15], as well as object detection and tracking pipelines based on You Only Look Once (YOLO), Deep Simple Online and Realtime Tracking (DeepSORT), and optical flow for fish counting and population management [16]. While these methods demonstrate promising performance in controlled or low-density environments, they often rely on heuristic association strategies and exhibit frequent identity switches and fragmented trajectories under dense occlusions and fast motion, limiting their suitability for continuous and fine-grained behavioral analysis [17]. Although these recent approaches provide valuable benchmarks, they are mostly limited to low-density or controlled conditions and do not fully address the challenges of dense aquaculture environments with frequent occlusions, rapid non-rigid motion, and visually similar individuals.

More recently, general-purpose multi-object tracking (MOT) frameworks, including joint detection-and-tracking methods such as CenterTrack [18], tracking-by-detection approaches such as OC_SORT, and transformer-based trackers such as TransCenter, TPTrack, and MOTR, have achieved strong performance on terrestrial benchmarks. Several recent studies have attempted to apply or adapt these frameworks to aquatic or underwater scenarios. However, most existing MOT methods are primarily designed for rigid objects and relatively stable imaging conditions, and their direct application to underwater multi-fish scenarios remains suboptimal. Frequent occlusions, appearance ambiguity, and abrupt motion changes commonly observed in aquaculture environments challenge the robustness of existing motion modeling and data association strategies. Although sonar-based or multimodal systems can partially alleviate visual limitations, their lower spatial resolution, sensitivity to noise, and higher computational cost restrict their applicability in high-precision behavioral studies [19,20].

To address these challenges, we propose an improved CenterTrack-based framework for multi-fish tracking in dense aquaculture environments. The proposed method aims to produce stable and high-quality trajectories under realistic underwater conditions, providing a reliable technical foundation for downstream behavioral analysis. In addition, we introduce MF25 dataset, which consists of 75 individually annotated fish recorded under diverse lighting conditions, population densities, and occlusion scenarios, offering a realistic benchmark for evaluating multi-fish tracking performance in aquaculture settings.

To overcome the above limitations and improve the robustness of multi-fish tracking in realistic aquaculture environments, this work makes the following contributions:

(1) A multi-fish tracking framework based on CenterTrack is proposed for reconstructing and refining historical features, which can better adapt to dense occlusions and rapid, non-rigid fish motion and improve trajectory stability and reducing identity switches.
(2) Adaptive motion–appearance fusion and occlusion-aware trajectory recovery strategies are introduced to handle unreliable associations caused by fast motion, visual ambiguity, and prolonged occlusions. which can improve tracking robustness in realistic aquaculture environment.
(3) A self-built MF25 multi-fish tracking dataset is constructed, containing annotated video sequences of 75 fish captured in a unified underwater scene. It features diverse motion patterns and complex group interactions, providing frame-level detections and trajectory-level ground truth for systematic evaluation.
(4) Extensive experiments and ablation studies show that the proposed method achieves robust tracking performance, producing trajectories with improved continuity and identity consistency, suitable for downstream behavioral and group-dynamics analysis.

The remainder of this article is organized as follows: Section 2 describes the proposed methodology. Section 3 introduces the MF25 dataset and experimental setup and presents the experimental results and comparisons with baseline methods. Section 4 discusses limitations and potential applications. Section 5 concludes this study.

## 2. Materials and Methods

### 2.1. Framework

In practical industrialized fish farming environments, underwater video data often suffer from prolonged occlusions, dense fish aggregations, and severe morphological deformations due to rapid movement. These challenges significantly impair tracking accuracy, falling short of operational requirements. Additionally, farm-raised fish of the same species often exhibit highly similar appearances and body structures, making individual identification and multi-object tracking particularly difficult.

**Figure 1:**
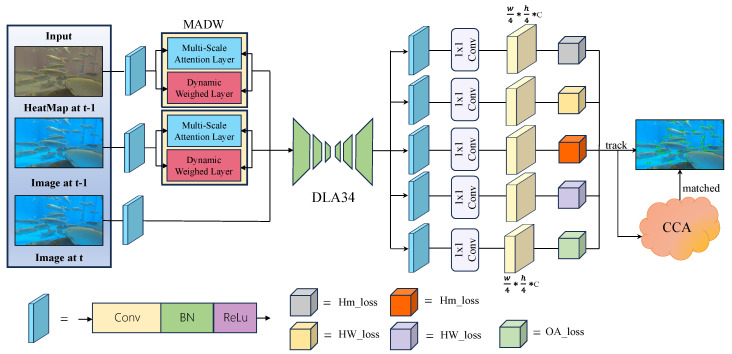
Overview of the proposed multi-fish tracking framework.

To address the above challenges, we propose an enhanced CenterTrack-based multi-fish tracking framework, whose system architecture is illustrated in Figure 1. The model takes three inputs: the current frame image $I_{t}$, the previous frame image $I_{t-1}$, and the previous frame’s heatmap features $H_{t-1}$. A novel Multi-scale Attention and Dynamic Weighting (MADW) module is introduced to process features extracted from $I_{t-1}$ and $H_{t-1}$, improving robustness against prolonged occlusions and severe non-rigid deformations. The refined historical features are then fused with features from $I_{t}$ to generate enriched spatial representations.

**Figure 2:**
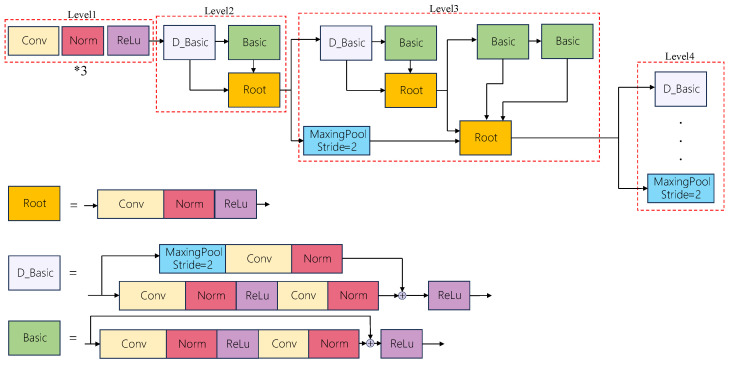
Structural Diagram of the DLA34 Neural Network.

These fused representations are fed into a Deep Layer Aggregation (DLA-34) backbone [21] for multi-scale feature extraction, as shown in Figure 2. In addition to the original output heads, we introduce an Occlusion-Aware Head (OAHead), which enhances edge-aware feature representations and promotes identity consistency for visually similar fish under heavy occlusion. During training, Focal Loss [22] and Dice Loss [23] are jointly employed with an adaptive weighting mechanism to balance positive and negative samples while improving boundary continuity. Finally, the processed features are passed to a Cascade Correspondence Association (CCA) algorithm, which performs a three-stage matching strategy to handle detections with varying confidence levels, enabling efficient trajectory update or termination for real-time online tracking.

### 2.2. Multi-Scale Attention and Dynamic Weighting

**Figure 3:**
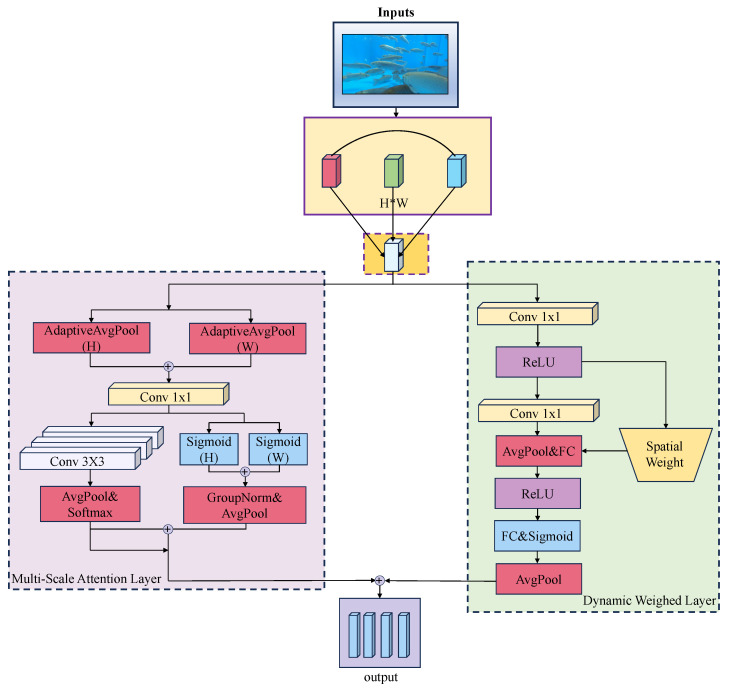
Architecture of the MADW module.

The MADW module refines input features using two complementary layers: Multi-scale Attention and Dynamic Weighting, shown in Figure 3.

The Multi-scale Attention layer adaptively fuses spatial and channel-wise features across multiple scales, improving both local and global contextual dependencies. Channel-grouped features undergo 1 × 1 and 3 × 3 convolutions to capture fine-grained and contextual information, respectively. Horizontal pooling reduces spatial dimensionality while preserving structural cues. The pooled features are concatenated and further fused using a 1 × 1 convolution, followed by Sigmoid activation to emphasize critical channels and refine local features. This design significantly enhances robustness against rapid movement and severe occlusions by exploiting multi-scale information from preceding frames.

The Dynamic Weighting layer adjusts the importance of different feature branches in real time. It employs three parallel convolutional branches (1 × 1, 3 × 3, and 5 × 5) to extract features with varying receptive fields, along with global average pooling for dimensionality reduction. These multi-scale features are concatenated and fused via a 1 × 1 convolution to generate enhanced representations. This mechanism adaptively adjusts the fusion weights between historical heatmap features and current frame features, effectively accommodating scale and positional variations while suppressing redundant information and emphasizing key regions. Together, these two layers improve the model’s perception in challenging underwater tracking scenarios.

### 2.3. Occlusion-Aware Head

The OAHead processes DLA-34 backbone outputs through two branches aimed at occlusion prediction and feature rectification. Each branch uses parallel 3 × 3 and 1 × 1 convolutions; the occlusion branch further applies 1 × 1 convolution for dimensionality reduction, while the rectification branch employs Instance Normalization to retain instance-level details.

**Figure 4:**
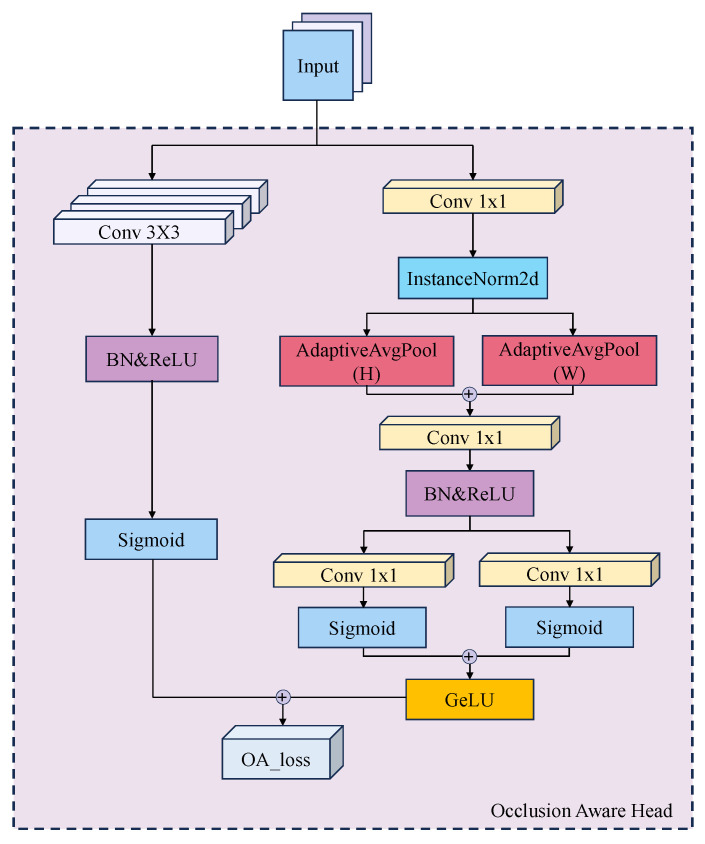
Architecture of the OAHead module.

To capture global spatial features without positional loss, horizontal and vertical pooling are applied, followed by concatenation and 1 × 1 convolution for efficient dimension reduction. Separable 1 × 1 convolutions learn independent attention weights along horizontal and vertical directions. Both branches use Sigmoid activations for soft attention and GeLU for nonlinear enhancement, extracting features that are robust to occlusion while suppressing irrelevant background noise, as shown in Figure 4.

To address class imbalance, the module employs a standard Focal Loss:

(1) $$L_{\mathrm{focal}}={\left(1-p_{t}\right)}^{2}\;\cdot \;\left[-y_{t}\log\left(p_{t}\right)-\left(1-y_{t}\right)\log\left(1-p_{t}\right)\right]$$

where $p_{t}$ is the predicted occlusion probability, $y_{t}$ the ground-truth label, and ${\left(1-p_{t}\right)}^{2}$ the hard-sample modulation factor. Dice Loss is added to maximize the IOU between predicted and true masks, mitigating pixel-level imbalance:

(2) $$L_{\mathrm{dice}\;}=1-\frac{2\sum \left(p_{t}\;\cdot \;y_{t}\right)+\epsilon }{\sum p_{t}+\sum y_{t}+\epsilon }$$

An adaptive weighting mechanism dynamically adjusts α (the Focal Loss weight) and β (the Dice Loss weight) based on gradient directions, ensuring a balanced contribution from each term. The total loss is thus:

(3) $$\left\{\begin{array}{c} \alpha_{\mathrm{new}}=\alpha \;\cdot \;\mathrm{sign}\left(\nabla_{\alpha }L_{\mathrm{current}}+10^{-8}\right)\\ \beta_{\mathrm{new}}=\beta \;\cdot \;\mathrm{sign}\left(\nabla_{\beta }L_{\mathrm{current}}+10^{-8}\right) \end{array}\right.$$

where $w_{\mathrm{base}}$ is the initial occlusion-loss weight. This multi-loss synergy enables the model to handle severe occlusions (via Focal Loss) while preserving precise boundary delineation (via Dice Loss), resulting in robust occlusion-aware feature learning.

(4) $$L_{\mathrm{total}}=w_{\mathrm{base}}\;\cdot \;\left(\alpha \;\cdot \;L_{\mathrm{focal}}+\beta \;\cdot \;L_{\mathrm{dice}}\right)$$

### 2.4. Cascade Correspondence Association

The proposed CCA fuses Kalman filtering with a three-stage cascade matching pipeline to robustly associate predicted tracks with incoming detections. The detailed execution flow of the proposed method is formally presented in Algorithm 1.

As illustrated in the algorithm, the procedure accepts the current frame’s detections as input and initializes the matching process. The core association mechanism (Lines 3–5) iterates through a defined sequence of three affinity metrics: Motion consistency, IoU overlap, and Euclidean Distance. In the first stage, the algorithm computes the Mahalanobis distance between Kalman-predicted states and detection measurements to handle motion consistency. Unmatched pairs then proceed to the IoU matching stage, which accommodates non-rigid deformations typical of swimming fish. Finally, any remaining ambiguous tracks undergo distance matching based on centroid proximity. This function Match extracts unmatched pairs, computes the specific affinity matrix for the current stage, and solves the assignment problem using the Hungarian algorithm.
**Algorithm 1** Triple-Stage Cascade Matching  1:**procedure** Step(detections)  2:    matched←∅, output←[]  3:    **for stage** ∈ {Motion, IoU, Distance} **do**  4:          matches← Match(stage,detections,tracks,matched)  5:          Update matched,output with matches  6:    **end for**  7:    **for** unmatched det∈detections **do**  8:          **if** $\det.\mathrm{score}>\theta_{\mathrm{new}}$ **then**  9:                Initialize new track, add to output10:          **end if**11:    **end for**12:    **for** unmatched track∈tracks **do**13:          **if** track.age<max_age **then**14:                Propagate with motion model, decay score, add to output15:          **end if**16:      **end for**17:      **return** output18:**end procedure**19:**function** Match(stage, detections, tracks, matched)20:      Extract unmatched pairs21:      Compute affinity (motion/IoU/distance)22:      Solve with Hungarian algorithm23:      **return** valid matches24:**end function**

Following the cascade matching, the algorithm manages the lifecycle of the trajectories. As detailed in Lines 6–10, detections that remain unmatched after all three stages are treated as potential new targets; if their confidence score exceeds $\theta_{\mathrm{new}}$, a new track is initialized. Conversely, Lines 11–15 handle existing tracks that failed to match any detection. These tracks are propagated using the motion model to predict their position in the next frame. However, their confidence scores are decayed, and if a track remains unmatched for a period exceeding max_age, it is considered lost and purged from the memory to prevent false-positive drift.

**Figure 5:**
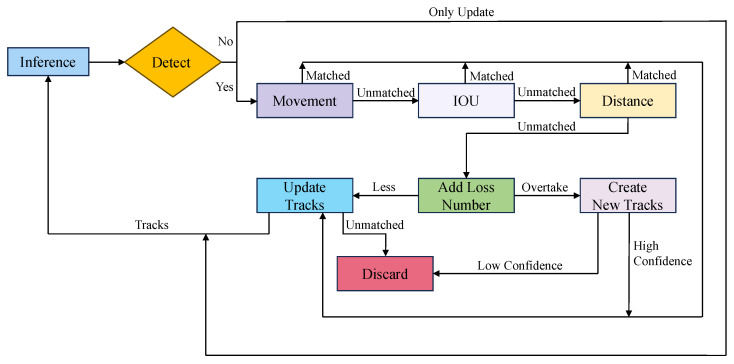
Architecture Flowchart of the Proposed CCA Triple Cascaded Matching Mechanism.

By cascading these three complementary criteria as structured in Algorithm 1, CCA strikes a balance between accuracy and efficiency, delivering robust, real-time multi-object tracking performance as shown in Figure 5.

### 2.5. Evaluation Metrics

To comprehensively evaluate the performance of multi-fish tracking, we adopt a set of standard multi-object tracking metrics. These metrics cover identity-level accuracy, detection precision, and trajectory completeness, strictly adhering to the standard definitions used in state-of-the-art literature [24,25].

We employ **Identification F1-score (IDF1)** and **Multiple Object Tracking Accuracy (MOTA)** as the primary evaluation metrics.

**IDF1** measures the consistency between predicted and ground-truth identities. It is essentially the **F1-score** calculated based on identity-level matching and is defined as follows:

(5) $$\mathrm{IDF}1=\frac{2\;\mathrm{IDTP}}{2\;\mathrm{IDTP}+\mathrm{IDFP}+\mathrm{IDFN}}$$

where IDTP (Identification True Positives), IDFP (Identification False Positives), and IDFN (Identification False Negatives) denote the global counts of correctly identified detections, false identifications, and missed identifications, respectively.

**MOTA** provides a holistic measure of tracking accuracy by jointly accounting for detection errors and identity switches:

(6) $$\mathrm{MOTA}=1-\frac{\mathrm{FN}+\mathrm{FP}+\mathrm{IDSW}}{\mathrm{GT}}$$

where FN (False Negatives), FP (False Positives), and IDSW (Identity Switches) represent the total counts of errors summed across all frames, and GT is the total number of ground-truth objects.

For a more detailed evaluation of identity consistency, we report **Identification Precision (IDP)** and **Identification Recall (IDR)** independently:

(7) $$\mathrm{IDP}=\frac{\mathrm{IDTP}}{\mathrm{IDTP}+\mathrm{IDFP}}$$

(8) $$\mathrm{IDR}=\frac{\mathrm{IDTP}}{\mathrm{IDTP}+\mathrm{IDFN}}$$

To evaluate trajectory continuity, we adopt **Mostly Tracked (MT)** and **Mostly Lost (ML)** with separate definitions:

(9) $$\mathrm{MT}=\frac{N_{\mathrm{MT}}}{N_{\mathrm{GT}\_\mathrm{traj}}}$$

(10) $$\mathrm{ML}=\frac{N_{\mathrm{ML}}}{N_{\mathrm{GT}\_\mathrm{traj}}}$$

where $N_{\mathrm{GT}\_\mathrm{traj}}$ is the total number of ground-truth trajectories, $N_{\mathrm{MT}}$ and $N_{\mathrm{ML}}$ denote the number of trajectories tracked for at least 80% and less than 20% of their lifespan, respectively.

Finally, **Higher Order Tracking Accuracy (HOTA)** [25] provides a unified evaluation of detection and association:

(11) $$\mathrm{HOTA}=\sqrt{\mathrm{DetA}\;\cdot \;\mathrm{AssA}}$$

where **Detection Accuracy (DetA)** and **Association Accuracy (AssA)** are defined using global tracking components:

(12) $$\mathrm{DetA}=\frac{\mathrm{TP}}{\mathrm{TP}+\mathrm{FP}+\mathrm{FN}}$$

(13) $$\mathrm{AssA}=\frac{1}{\left|\mathrm{TP}\right|}\sum_{c\in \mathrm{TP}}A\left(c\right)$$

Here, TP,FP,FN denote the global counts of detection-level true positives, false positives, and false negatives (where GT=TP+FN). For association, A(c) represents the association score for each matched detection *c*.

### 2.6. Training and Inference

The proposed framework has two distinct phases: **offline training** and **online inference**.

All video frames used for training were collected under consistent lighting and water turbidity conditions, ensuring uniform image quality and avoiding potential biases due to environmental variations.

During training, the network learns robust features for fish detection, occlusion handling, and trajectory association. Annotated video data are used to update all model parameters, minimizing Focal Loss, Dice Loss, and adaptive weighting mechanisms. This process is performed offline, usually on high-end GPUs.

During inference, the trained model is applied to new video frames in real-time. No parameters are updated; the model performs forward passes only. Modules such as MADW, OAHead, and CCA continue to refine features, enhance occlusion robustness, and associate tracks across frames. In this phase, the focus is on fast and accurate processing of incoming video data.

Although actual performance depends on hardware and input resolution, the model can typically achieve 30 FPS on Powerful GPUs such as the NVIDIA RTX 4090, with memory usage around 6–8 GB per frame. These numbers suggest that the system is suitable for industrial deployment in aquaculture, balancing both accuracy and computational efficiency.

## 3. Experiments

### 3.1. DataSet

**Figure 6:**
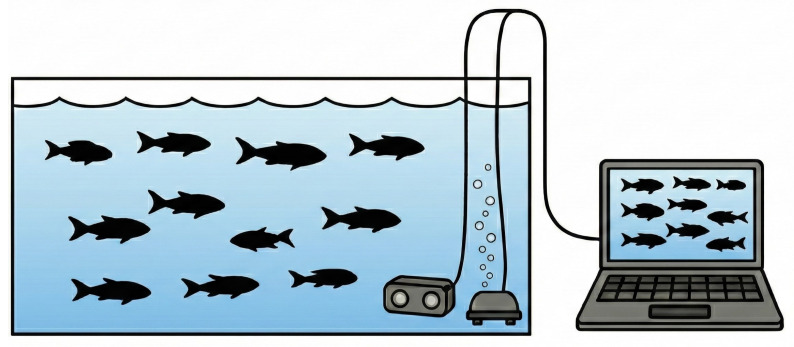
The culturing tank at the experimental platform.

**Figure 7:**
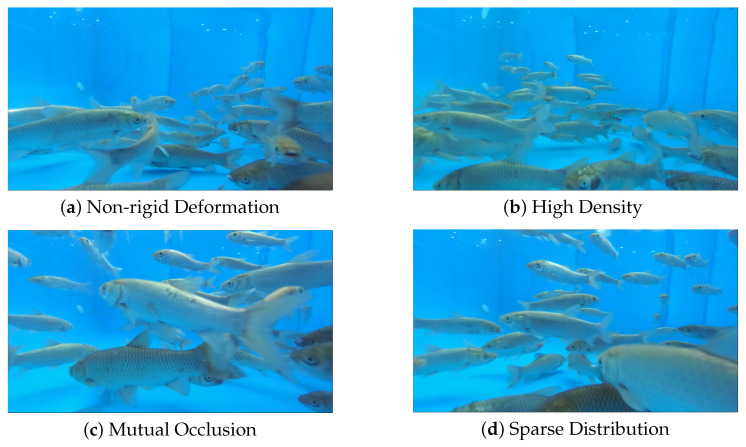
These 4 pictures are samples from the MF25 dataset.

MF25 is a multi-fish tracking dataset consisting of short video clips, each containing 25 consecutive frames, recorded in aquaculture tanks with up to 75 simultaneously visible fish. The dataset was collected during December 2024 at South China Agricultural University in Guangzhou, China. The experimental configuration comprised a 2.5 m (Length) × 1.5 m (Width) × 0.9 m (Height) aquaculture tank stocked with 75 grass carp (Ctenopharyngodon idellus) ranging from 8 to 25 cm in total length (including caudal fin), simulating industrial aquaculture conditions. All experiments follow common aquaculture practice, where each tank contains only a single fish species, and tracking is performed within the same enclosure. The tank incorporated an aerator and environmental monitoring systems to maintain optimal aquatic conditions and track growth parameters, as shown in Figure 6. A Shanghai Huachuan Technologies ZED 2i IP66-rated stereo camera (Shanghai, China)—engineered for harsh environment operation and suitable for agricultural, industrial, and pharmaceutical applications—was deployed. Specifically, the camera was explicitly configured to record at a resolution of 1920 × 1080 pixels and a frame rate of 30 frames per second (FPS) to ensure high-definition data acquisition, as shown in Figure 7 and Table 1.

The recorded footage exhibits frequent severe occlusion instances and morphological deformations, with subjects occasionally exiting the frame due to camera perspective constraints. Schooling behavior predominates in most sequences, inducing rapid locomotion, while residual footage contains slow-moving specimens susceptible to persistent occlusion. We segmented 10–15 s clips into 1917 training frames and 350 test frames. Each frame was annotated by a single expert; inter-annotator agreement was not applicable. All sequences were annotated following the Multiple Object Tracking Challenge (MOTChallenge) format specifications.

Experimental trials used a deep learning server equipped with an NVIDIA RTX 4090 graphics processing unit (GPU), an Intel Xeon Gold 6338 central processing unit (CPU), and 128 GB random access memory (RAM). The framework was implemented in PyTorch 1.13.1+cuda 11.7 with the following parameters: 512 × 512 input resolution, batch size 8, learning rate 1.25 × 10^−4^, dropout rate 0.1, and 70 training epochs. During tracking, detection thresholds were set to track_thresh = 0.4 and pre_thresh = 0.4, with trajectories failing to match for over 3 consecutive frames were discarded.

### 3.2. Comparative Study

To evaluate the performance of ours method in multi-fish underwater tracking, we conducted comprehensive comparisons with two tracking paradigms:joint detection and embedding (JDE) and separate detection and embedding (SDE) and other relevant models. All experiments were performed on the underwater MF25 dataset to validate our model’s efficacy in complex multi-fish tracking scenarios.

**Figure 8:**
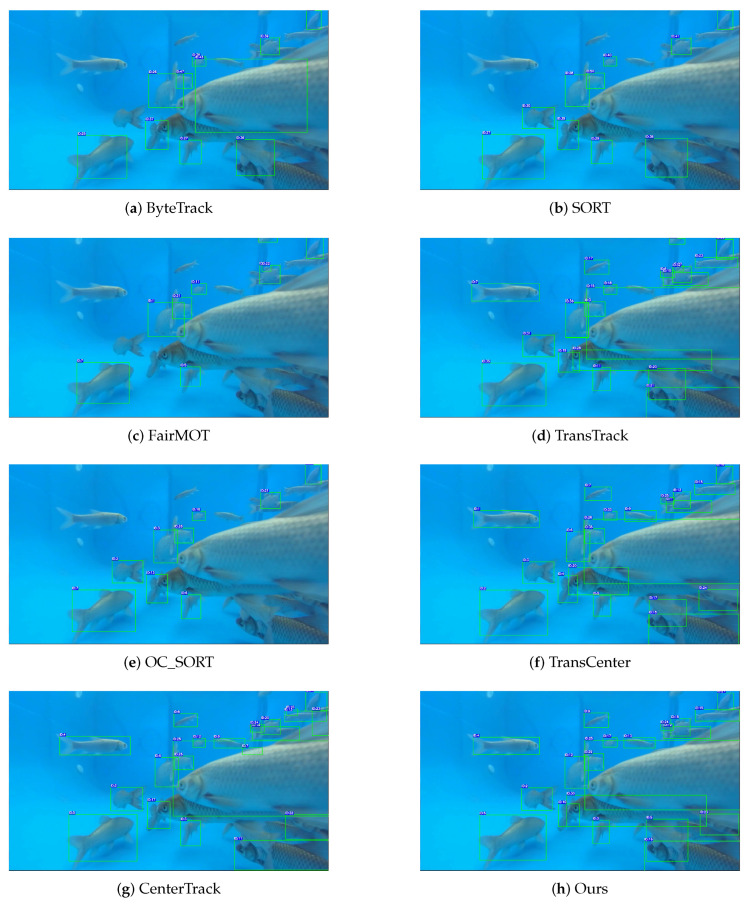
Trackingresults of different methods on the MF25 dataset, demonstrating their performance in underwater scenarios. Each fish is annotated with a distinct numeric ID and a colored bounding box.

To demonstrate the performance of ours method for complex underwater multi-fish tracking, we compared it against classic algorithms including SORT [26], DeepSORT [27,28], ByteTrack [29], OCSORT [30], CenterTrack, MOTR [31], FairMOT [32], TransCenter [33], TransTrack [34], TPTrack [35] and Ours Method using the specified metrics. All results were obtained by training and tracking on the MF25 dataset to evaluate detection accuracy and tracking capability for the same targets.shown in Figure 8.

Given the frequent target loss and identity switches characteristic of fish objects, the IDF1 metric was employed as a critical measure of identity-preserving tracking performance, while the MOTA metric was used to assess overall multi-object tracking accuracy. As shown in Table 2, the SORT method is suitable for real-time systems, while DeepSORT improves upon SORT by enhancing occlusion handling. ByteTrack combines the strengths of SORT and DeepSORT and demonstrates particular effectiveness in extremely crowded scenarios. OCSORT excels in high-speed motion scenes and shows advantages for tracking fast-moving underwater fish. FairMOT offers robust recognition in dense scenes, effectively addressing underwater crowding challenges compared to SORT and DeepSORT approaches. TransTrack provides a lightweight solution that achieves SORT-like speed while maintaining good accuracy. TransCenter achieves significant improvements in long-range temporal association, substantially boosting performance for underwater multi-fish tracking. Unlike TransCenter, MOTR offers stronger handling of fish deformation and occlusion. TPTrack represents the performance leader among non-Transformer models, significantly reducing computational overhead and resource consumption.

Our proposed method significantly enhances the accuracy and robustness of inter-frame association in multi-fish tracking tasks. Specifically, by introducing the MADW module, the model effectively strengthens feature representation during inter-frame feature extraction, thereby increasing the information density and discriminative capability of extracted features. This improvement provides a more reliable foundation for subsequent matching and tracking processes.

In addition, the designed OAHead module and the improved loss function effectively reduce misidentification and identity confusion among different fish under severe occlusion and high-density crowding scenarios. These targeted enhancements substantially improve the model’s robustness and generalization ability in complex underwater environments.

Moreover, the proposed CCA algorithm further improves identity preservation during the tracking process. It enables the tracker to avoid identity switches and target losses even when fish interact frequently, undergo large pose changes, or experience partial occlusions, ensuring more consistent and continuous trajectories.

The comprehensive experimental results demonstrate that our approach demonstrates competitive or superior performance across multiple metrics compared with Transformer-based methods such as TransCenter and TPTrack in multi-fish tracking scenarios. Compared with the computationally intensive TransTrack, our method achieves a favorable balance between tracking accuracy and model complexity.

Among all evaluated metrics, our improved method achieves the highest scores in both MOTA and IDF1, clearly validating its superior performance and strong robustness in complex multi-fish tracking environments. These results indicate that our framework not only surpasses existing approaches in accuracy but also exhibits remarkable advantages in model efficiency and practical applicability.

To further demonstrate robustness, the publicly available Uniform and Deformable Multi-fish Tracking (UD-MFT) dataset [36], which contains both underwater and above-water fish tracking sequences. To comprehensively evaluate the tracking performance, we compared the proposed method with a series of representative multi-object tracking algorithms, including SORT, DeepSORT, ByteTrack, FairMOT, CenterTrack, TransTrack, GTR [37], OCSORT, and TPTrack. As shown in Table 3, OCSORT achieved the highest IDF1 and IDR scores, while TPTrack obtained the best MOTP. Our improved CenterTrack framework (“Ours”) achieved the highest IDP and competitive overall performance, demonstrating the effectiveness of the proposed enhancements in multi-fish tracking. Our method achieves superior performance in both DetA and MOTA metrics. This demonstrates our model’s excellence in both single-frame detection capability and cross-sequence association ability, establishing it as a well-balanced and powerful approach that performs exceptionally in both core aspects of “detection” and “association”.

For the MF25 dataset, the model was trained for 70 epochs using the training split, while performance was monitored on a held-out validation set. Standard data augmentation techniques were applied during training, including random horizontal flipping, scale jittering, and color perturbation, to improve robustness to underwater appearance variations. Early stopping was not adopted; instead, the model checkpoint achieving the best validation performance was selected for final evaluation on the test set.

For the UDMFT dataset, the same training and validation protocol was followed to ensure consistency. The model was fine-tuned for 70 epochs using the official training split, with identical data augmentation settings. Model selection was based on validation performance, and all evaluations were conducted under the same evaluation metrics and protocols as those used for MF25.

The original resolution for all models was configured according to their backbone network requirements, ensuring that no model’s performance was compromised by resolution issues. In Table 4, we compared different backbone networks, providing detailed analysis of DLA, MobileNetV2 [38] and ResNet [39] across various dimensions. The results clearly indicate that DLA34 delivers the best performance among all tested backbone networks. All other parameters maintained their original default configurations.

### 3.3. Ablation Study and Analysis

**Figure 9:**
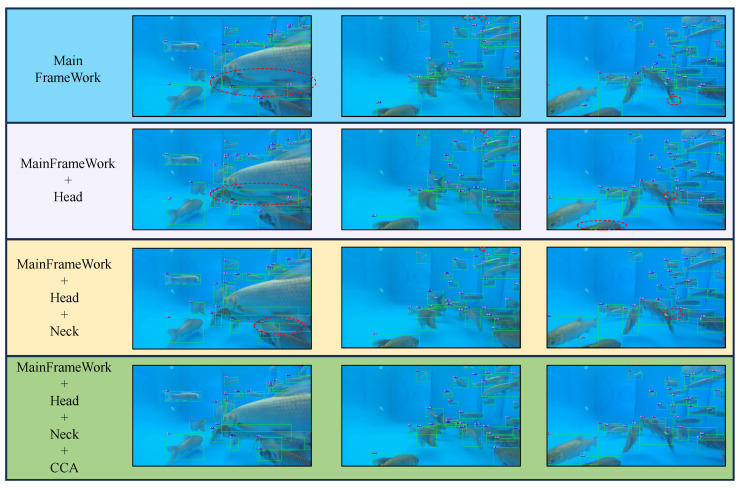
Tracking Results of Different Module Combinations at T, T + 50, and T + 150 Moments (T = 0, in Frames per Second), From **left** to **right**: T, T + 50, and T + 150 respectively. The dotted circles indicate missed detections where the tracking algorithm failed to identify the fish individuals.

Ablation studies were conducted on our enhanced CenterTrack framework in Table 5. Integration of the proposed MADW module into the three-branch input elevated performance to 78.9% IDF1, 83.9% MOTA, 81.6% IDP, 76.3% IDR, and 20.2% MOTP, confirming its efficacy in feature extraction enhancement. Subsequent incorporation of the OAHead module improved occlusion handling and edge discrimination through its specialized loss function, which optimizes positive-negative sample balance in occluded regions, enhances prediction-ground truth IOU alignment, and dynamically regulates loss contributions without manual intervention. This configuration achieved 79.0% IDF1, 81.7% MOTA, 81.8% IDP, 76.3% IDR, and 20.3% MOTP. shown in Figure 9.

Combined implementation of MADW and OAHead modules yielded 79.8% IDF1, 85.0% MOTA, 81.9% IDP, 77.8% IDR, and 20.0% MOTP. Final integration of our CCA method employing three-stage cascade matching (motion correlation, IOU similarity, distance association) to reconcile predicted and observed trajectories—enabled recovery of occluded and deformed targets, achieving peak performance of 82.5% IDF1, 85.8% MOTA, 84.7% IDP, 80.4% IDR, and 20.0% MOTP. These experiments collectively validate each module’s contribution to enhanced detection, tracking, and association capabilities.

### 3.4. Hyperparameter Optimization

We conducted hyperparameter experiments based on the improved CenterTrack framework. This study compared five key parameters: Track_Thresh, Pre_Thresh, BatchSize, Learning_Rate, and Epoch.

**Figure 10:**
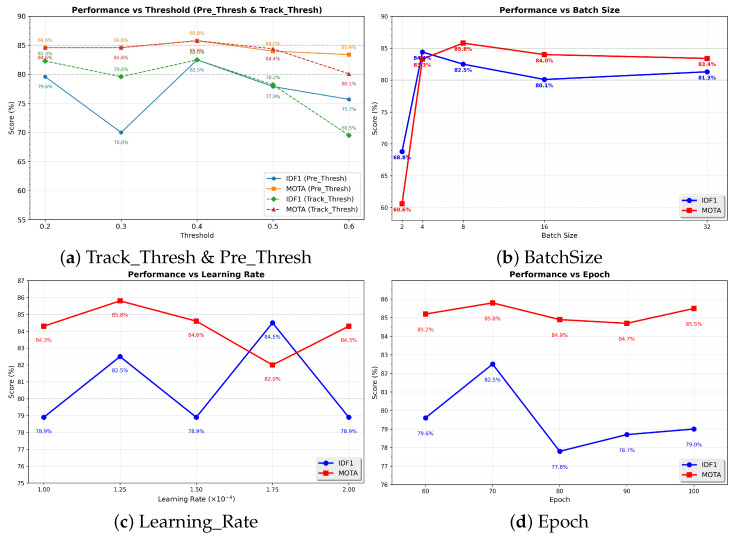
Hyperparameter Influence on Tracking Performance (IDF1 & MOTA) in the Improved CenterTrack Multi-Fish Method.

The results in Figure 10 demonstrate that setting both Track_Thresh and Pre_Thresh to 0.4 yields optimal network performance. At these values, the model achieves its highest scores: 82.5% IDF1, 85.8% MOTA, 84.7% IDP, 80.4% IDR, and 20% MOTP. Alternative parameter configurations resulted in varying degrees of performance degradation across these metrics.

For BatchSize, we evaluated settings of 2, 4, 8, 16, and 32. Performance metrics decreased slightly at BatchSize = 2, while values at 4, 16, and 32 remained comparable. This indicates that increasing BatchSize beyond 16 provides negligible performance gains. Given that BatchSize = 32 requires dual RTX 4090 GPUs for execution, we selected BatchSize = 8 as our final parameter to balance computational efficiency with hardware compatibility while maintaining peak performance.

Regarding the learning rate, experiments were conducted with values of 1 × 10^−4^, 1.25 × 10^−4^, 1.5 × 10^−4^, 1.75 × 10^−4^, and 2 × 10^−4^. The best performance was achieved at a learning rate of 1.25 × 10^−4^, which is likely due to faster and more stable convergence during training.

Epochs were evaluated at 60, 70, 80, 90, and 100. As shown in Figure 10, tracking performance improves steadily from 60 to 70 epochs, indicating progressive model convergence. The best performance is achieved at 70 epochs. When training is extended beyond 70 epochs, both IDF1 and MOTA exhibit a gradual decline, suggesting overfitting to the training data. Therefore, 70 epochs represent an optimal trade-off between sufficient convergence and generalization performance, and all experiments in this study were conducted using 70 training epochs.

**Figure 11:**
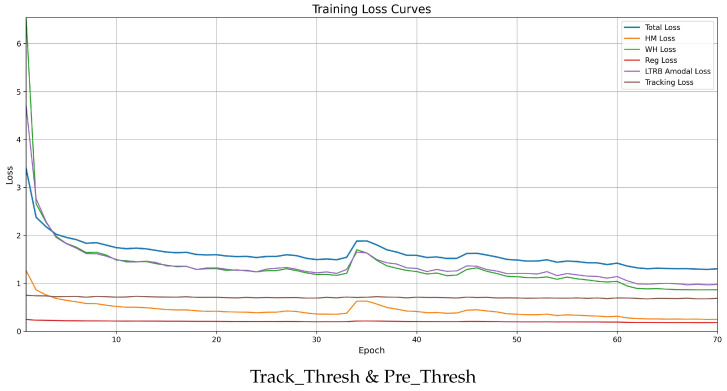
Training loss curves showing the convergence of total loss and individual components (HM Loss, WH Loss, Reg Loss, LTRB Amodal Loss, and Tracking Loss) over 70 epochs.

Figure 11 shows the training loss curves of the proposed method over 70 epochs, including the total loss and individual loss components, which consistently decrease and converge without significant oscillation, indicating stable and balanced optimization. The loss curves become relatively stable after around 50 epochs, suggesting that training for 70 epochs is sufficient to ensure convergence while avoiding unnecessary computational cost.

For a fair comparison, all baseline tracking methods were retrained on the MF25 training set using the same data split as the proposed method. Pre-trained weights provided by the original authors were used only for initialization, and all models were fine-tuned on MF25 to adapt to the underwater aquaculture scenarios.

Hyperparameters for each baseline were selected following the recommendations in their original papers and adjusted within a limited range to achieve stable convergence on MF25. No dataset-specific tuning beyond standard practices was performed, and all methods were evaluated under identical training and evaluation protocols.

## 4. Discussion

### 4.1. Key Features and Contributions

The proposed framework supports efficient multi-fish tracking while also estimating activity levels and group dynamics. By quantifying trajectory length and aggregate activity, the approach enables more precise assessment of fish vitality and adaptability to environmental changes. The method also mitigates the impact of water flow and other environmental disturbances on activity measurements, increasing the likelihood that observed motion reflects true fish behavior rather than imaging artifacts.

Ablation studies and comparative experiments demonstrated that the ours model maintains high tracking accuracy while preserving computational efficiency suitable for online tracking. This ensures that subsequent analyses, such as activity evaluation and trajectory assessment, are based on reliable and continuous tracking data. Visualization of group trajectories further confirms that the method captures group-level behavior accurately, supporting applications in behavioral analysis and aquaculture management.

### 4.2. Correlation Between Tracking Accuracy and Activity Estimation

**Figure 12:**
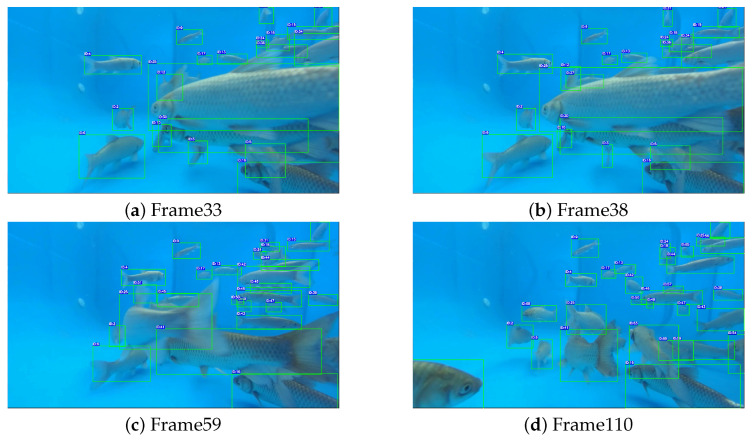
Tracking results of Multi-Fish Tracking Method Based on improved CenterTrack on the MF25 dataset, Verification of high accuracy and stable short-term tracking across diverse scenarios. Each fish is annotated with a distinct numeric ID and a colored bounding box.

Our analysis revealed that errors in activity estimation mainly arise from identity switches or missed tracking events. shown in Figure 12, the mean absolute percentage error (MAPE) per frame for both trajectory length and activity levels is inversely correlated with tracking metrics such as IDF1 and MOTA. This indicates that the reliability of activity measurements depends critically on robust identity preservation and continuous trajectory tracking.

### 4.3. Limitations and Future Directions

Although the proposed framework demonstrates strong tracking performance in controlled aquaculture environments, its scope and applicability should be interpreted with appropriate caution.

(1) **Environmental diversity and domain shift:** Experiments were conducted in relatively controlled tank environments that simulate industrial aquaculture. Although these settings capture realistic occlusion and crowding patterns, environmental diversity remains limited. Variations in illumination, water turbidity, camera viewpoints, and background complexity in real production facilities may introduce domain shifts, potentially degrading tracking performance when the model is directly applied without further adaptation.
(2) **Identity persistence under long-term occlusion:** The proposed tracker maintains short-term visual identities rather than persistent biological identities. When fish leave the field of view or experience prolonged occlusions, identity consistency may be compromised. This limitation is particularly relevant for long-term behavioral or individual-based studies, where identity switches may affect the interpretation of fine-grained behavioral patterns.
(3) **Scope of behavioral analysis:** The current study primarily focuses on trajectory-based indicators such as activity level and movement length. While these metrics are informative, they do not fully capture complex group dynamics or physiological states. Future work should investigate richer behavioral descriptors and their relationships with fish health, stress, and environmental variables.
(4) **Computational constraints and deployment:** Although a systematic analysis of computational efficiency across different hardware platforms was not conducted, the proposed framework achieves real-time tracking under the current experimental configuration with 30 FPS input video on a single RTX 4090 GPU. The introduced multi-stage association and motion modeling components are lightweight and do not substantially increase computational complexity compared to the baseline CenterTrack framework. Nevertheless, deployment on resource-constrained edge devices remains challenging due to the deep backbone. Future work will focus on model compression, backbone optimization, and lightweight association strategies to further improve deployment flexibility.
(5) **2D imaging constraints and depth ambiguity:** The proposed framework is evaluated under a monocular 2D imaging setup, which inherently limits observability along the depth axis. Fish moving outside the camera field of view or overlapping in depth cannot be continuously tracked, and depth information from the stereo setup was not explicitly exploited in this study. These factors constrain long-term continuous monitoring and should be addressed in future work through multi-view or depth-aware extensions.

Future research will extend the dataset to environments with higher fish densities and more complex visual conditions to improve robustness under domain shifts. Incorporating re-identification features and long-term temporal modeling will be important for preserving identity continuity in extended observations. In addition, optimizing software and hardware configurations will be essential for scalable, real-time deployment in practical aquaculture systems.

### 4.4. Implications for Aquaculture and Behavioral Analysis

The proposed framework provides a practical tool for quantitative behavioral analysis in aquaculture. By combining accurate multi-fish tracking with reliable activity estimation, it enables:

(1) Non-invasive monitoring of fish vitality and adaptability to environmental changes.
(2) Real-time assessment of group behavior and schooling patterns.
(3) Data-driven support for feeding strategies, health management, and tank design.

Overall, this method offers a foundation for automated aquaculture monitoring and future studies on fish behavior, bridging the gap between advanced tracking technology and practical aquaculture applications.

## 5. Conclusions

This study introduced an enhanced CenterTrack-based framework for multi-fish tracking, which was designed to generate high-quality and continuous trajectories in dense underwater environments with frequent occlusions and non-rigid fish motion. The proposed framework effectively addressed challenges such as temporary target loss, appearance similarity, and scale variation, enabling stable identity preservation across frames.

To evaluate its performance, we constructed MF25, a real-world underwater multi-fish dataset containing 75 individually annotated grass carp recorded under varied conditions, including occlusions, varying illumination, and high population density. On MF25, the proposed approach achieved an IDF1 score of 82.5%, a MOTA of 85.8%, and an IDP of 84.7%, demonstrating robust identity consistency and accurate multi-fish tracking performance.

These results indicated that the framework reliably produced high-precision trajectories suitable for downstream behavioral analyses, including swimming pattern characterization, social interaction analysis, and responses to environmental changes. While performance under extreme low-light conditions and deployment on resource-constrained devices remained a limitation, the framework provided a practical tool for behavior-driven research and intelligent monitoring in industrial aquaculture systems. Future work would focus on domain adaptation, re-identification features, and model compression to broaden applicability.

## Figures and Tables

**Table 1:** Raw video clips of MF25 dataset.

ID	Name	Image Size	Sequence Length	Frame Rate	Format
1	Severely Deformed-01	1920 × 1080 pixels	177	30	PNG
2	Severely Deformed-02	1920 × 1080 pixels	259	30	PNG
3	Multi-Density-01	1920 × 1080 pixels	265	30	PNG
4	Multi-Density-02	1920 × 1080 pixels	193	30	PNG
5	Severe Occlusion-01	1920 × 1080 pixels	247	30	PNG
6	Normal-01	1920 × 1080 pixels	535	30	PNG
7	Normal-02	1920 × 1080 pixels	601	30	PNG

**Table 2:** Comparison with Other Multiple Object Tracking Models on MF25 Dataset.

Method	IDF1 (%)	IDP (%)	IDR (%)	MOTA (%)	MOTP	MT	ML
SORT	51.5	**94.8**	35.3	35.6	**0.18**	4	36
DeepSORT	52.3	94.3	36.2	35.8	0.20	8	33
ByteTrack	52.2	94.3	36.1	35.7	0.20	8	34
OCSORT	49.7	95.6	33.6	33.6	0.18	3	39
FairMOT	47.8	88.0	32.9	33.5	0.24	6	35
CenterTrack	75.5	78.3	73.0	83.1	0.19	51	2
TransCenter	74.0	76.4	71.9	84.6	0.19	52	**1**
TransTrack	67.3	68.8	65.9	78.7	0.19	50	**1**
MOTR	75.6	83.5	69.1	79.4	0.20	20	14
TPTrack	54.1	94.6	37.8	37.7	0.18	9	33
Ours	**82.5**	84.7	**80.4**	**85.8**	0.19	**55**	**1**

MT = Mostly Tracked trajectories, ML = Mostly Lost trajectories. Note: Bold values indicate the best performance.

**Table 3:** The Comparison with other Multiple Object Tracking Models In UD-MFT.

Method	HOTA (%)	DetA (%)	AssA (%)	MOTA (%)	IDF1 (%)
SORT	32.8	56.2	19.3	66.7	30.6
DeepSORT	25.6	61.3	10.8	69.6	21.5
ByteTrack	35.6	52.2	24.8	56.4	38.1
FairMOT	16.3	29.0	9.4	31.4	15.5
CenterTrack	27.0	74.3	11.0	69.1	39.1
TransTrack	33.5	76.3	16.5	71.3	41.9
GTR	30.9	49.8	19.4	60.1	32.9
OCSORT	**39.6**	55.3	**28.4**	70.8	46.7
TPTrack	33.8	58.2	21.8	54.1	**48.6**
Ours	29.5	**77.3**	13.4	**72.5**	42.4

HOTA = Higher Order Tracking Accuracy, DetA = Detection Accuracy, AssA = Association Accuracy. Note: Bold values indicate the best performance.

**Table 4:** The Comparison with other Backbone in Multi-Fish Tracking Method Based on improved CenterTrack.

Method	IDF1 (%)	IDP (%)	IDR (%)	MOTA (%)	MOTP (%)
DLA34	**82.5**	**84.7**	**80.4**	**85.8**	**0.19**
DLA60	81.3	83.8	79.0	82.9	0.20
DLA102	78.3	80.5	76.3	82.2	0.20
MobilenetV2	80.6	81.0	80.2	78.8	0.21
ResNet	79.4	80.1	77.5	79.2	0.21

Note: Bold values indicate the best performance.

**Table 5:** Ablation study on the Multi-Fish Tracking method based on improved CenterTrack.

Method	IDF1 (%)	IDP (%)	IDR (%)	MOTA (%)	MOTP
CenterTrack	75.5	78.3	73.0	83.1	0.19
+MADW	78.8	81.6	76.3	83.9	0.20
+OAHead	79.0	81.8	76.3	81.7	0.20
+CCA	78.4	79.6	77.1	83.7	0.20
+MADW + OAHead	79.8	81.9	77.8	85.0	0.20
+MADW + OAHead + CCA	**82.5**	**84.7**	**80.4**	**85.8**	**0.19**

Note: Bold values indicate the best performance.

## Data Availability

The data can be accessed at https://github.com/linmingrun/Underwater-Multi-Fish-Tracking-Method-Based-on-improved-CenterTrack.git (accessed on 1 October 2025).

## References

[1] Liu Y., Li B., Zhou X., Li D., Duan Q. (2024). FishTrack: Multi-object tracking method for fish using spatiotemporal information fusion. Expert Syst. Appl..

[2] Zhang H., Li W., Qi Y., Liu H., Li Z. (2023). Dynamic fry counting based on multi-object tracking and one-stage detection. Comput. Electron. Agric..

[3] Zhao H., Cui H., Qu K., Zhu J., Li H., Cui Z., Wu Y. (2024). A fish appetite assessment method based on improved ByteTrack and spatiotemporal graph convolutional network. Biosyst. Eng..

[4] Mei Y., Sun B., Li D., Yu H., Qin H., Liu H., Yan N., Chen Y. (2022). Recent advances of target tracking applications in aquaculture with emphasis on fish. Comput. Electron. Agric..

[5] Liu Y., Li B., Si L., Liu C., Li D., Duan Q. (2025). Group activity amount estimation for fish using multi-object tracking. Aquac. Eng..

[6] Liu Z., Wang X., Wang C., Liu W., Bai X. (2025). SparseTrack: Multi-object tracking by performing scene decomposition based on pseudo-depth. IEEE Trans. Circuits Syst. Video Technol..

[7] Cui M., Liu X., Liu H., Zhao J., Li D., Wang W. (2025). Fish tracking, counting, and behaviour analysis in digital aquaculture: A comprehensive survey. Rev. Aquac..

[8] Xi C., Cui M., Yin J., Gu H., Ouyang T., Feng J., Zeng L. (2025). Enhanced deep OC-SORT with YOLOv8-seg for robust fry tracking and behavior analysis in aquaculture. Aquaculture.

[9] Li Y., Tan H., Deng Y., Zhou D., Zhu M. (2025). Hypoxia monitoring of fish in intensive aquaculture based on underwater multi-target tracking. Comput. Electron. Agric..

[10] Yu S. (2022). Sonar image target detection based on deep learning. Math. Probl. Eng..

[11] Fu Z., Zhang S., Zhou L., Wang Y., Feng X., Zhao X., Sun M. (2024). Zebrafishtracker3D: A 3D skeleton tracking algorithm for multiple zebrafish based on particle matching. ISA Trans..

[12] Wei D., Bao E., Wen Y., Zhu S., Ye Z., Zhao J. (2021). Behavioral spatial-temporal characteristics-based appetite assessment for fish school in recirculating aquaculture systems. Aquaculture.

[13] Yao M., Huo Y., Tian Q., Zhao J., Liu X., Wang R., Xue L., Wang H. (2025). FMRFT: Fusion Mamba and DETR for query time sequence intersection fish tracking. Comput. Electron. Agric..

[14] Folkman L., Vo Q.L., Johnston C., Stantić B., Pitt K.A. (2025). A computer vision method to estimate ventilation rate of Atlantic salmon in sea fish farms. arXiv.

[15] Høgstedt E.B., Schellewald C., Mester R., Stahl A. (2025). Automated computer vision-based individual salmon (*Salmo salar*) breathing rate estimation (SaBRE) for improved state observability. Aquaculture.

[16] Khiem N.M., Van Thanh T., Dung N.H., Takahashi Y. (2025). A novel approach combining YOLO and DeepSORT for detecting and counting live fish in natural environments through video. PLoS ONE.

[17] Abangan A.S., Kopp D., Faillettaz R. (2023). Artificial intelligence for fish behavior recognition: Advances and applications. Front. Mar. Sci..

[18] Zhou X., Koltun V., Krähenbühl P. Tracking objects as points. Proceedings of the Computer Vision—ECCV 2020.

[19] Winkler J., Badri-Hoeher S., Barkouch F. (2023). Activity segmentation and fish tracking from sonar videos by combining artifacts filtering and a Kalman approach. IEEE Access.

[20] Kandimalla V., Richard M., Smith F., Quirion J., Torgo L., Whidden C. (2023). Automated Detection, Classification and Counting of Fish in Fish Passages with Deep Learning. Front. Mar. Sci..

[21] Yu F., Wang D., Shelhamer E., Darrell T. (2017). Deep layer aggregation. arXiv.

[22] Lin T.Y., Goyal P., Girshick R., He K., Dollár P. Focal loss for dense object detection. Proceedings of the 2017 IEEE International Conference on Computer Vision (ICCV).

[23] Li X., Sun X., Meng Y., Liang J., Wu F., Li J., Jurafsky D., Chai J., Schluter N., Tetreault J. (2020). Dice loss for data-imbalanced NLP tasks. Proceedings of the 58th Annual Meeting of the Association for Computational Linguistics.

[24] Luiten J. (2020). TrackEval. https://github.com/JonathonLuiten/TrackEval.

[25] Luiten J., Osep A., Dendorfer P., Torr P., Geiger A., Leal-Taixé L., Leibe B. (2020). HOTA: A higher order metric for evaluating multi-object tracking. Int. J. Comput. Vis..

[26] Bewley A., Ge Z., Ott L., Ramos F., Upcroft B. Simple online and realtime tracking. Proceedings of the 2016 IEEE International Conference on Image Processing (ICIP).

[27] Wojke N., Bewley A. Deep cosine metric learning for person re-identification. Proceedings of the 2018 IEEE Winter Conference on Applications of Computer Vision (WACV).

[28] Wojke N., Bewley A., Paulus D. (2017). Simple online and realtime tracking with a deep association metric. arXiv.

[29] Zhang Y., Sun P., Jiang Y., Yu D., Weng F., Yuan Z., Luo P., Liu W., Wang X. ByteTrack: Multi-object tracking by associating every detection box. Proceedings of the Computer Vision—ECCV 2022.

[30] Cao J., Pang J., Weng X., Khirodkar R., Kitani K. Observation-centric SORT: Rethinking SORT for robust multi-object tracking. Proceedings of the 2023 IEEE/CVF Conference on Computer Vision and Pattern Recognition (CVPR).

[31] Zeng F., Dong B., Zhang Y., Wang T., Zhang X., Wei Y. MOTR: End-to-end multiple-object tracking with transformer. Proceedings of the Computer Vision—ECCV 2022.

[32] Zhang Y., Wang C., Wang X., Zeng W., Liu W. (2021). FairMOT: On the fairness of detection and re-identification in multiple object tracking. Int. J. Comput. Vis..

[33] Xu Y., Ban Y., Delorme G., Gan C., Rus D., Alameda-Pineda X. (2021). TransCenter: Transformers with dense representations for multiple-object tracking. arXiv.

[34] Sun P., Cao J., Jiang Y., Zhang R., Xie E., Yuan Z., Wang C., Luo P. (2020). TransTrack: Multiple-object tracking with transformer. arXiv.

[35] Li Y., Zhao H., Liu Q., Liang X., Xiao X. (2024). TPTrack: Strengthening tracking-by-detection methods from tracklet processing perspectives. Comput. Electr. Eng..

[36] Huang J., Yu X., An D., Ning X., Liu J., Tiwari P. (2025). Uniformity and deformation: A benchmark for multi-fish real-time tracking in the farming. Expert Syst. Appl..

[37] Zhou X., Yin T., Koltun V., Krähenbühl P. Global Tracking Transformers. Proceedings of the 2022 IEEE/CVF Conference on Computer Vision and Pattern Recognition (CVPR).

[38] Sandler M., Howard A., Zhu M., Zhmoginov A., Chen L.C. (2019). MobileNetV2: Inverted residuals and linear bottlenecks. arXiv.

[39] He K., Zhang X., Ren S., Sun J. Deep residual learning for image recognition. Proceedings of the 2016 IEEE Conference on Computer Vision and Pattern Recognition (CVPR).

